# A New MEPED‐Based Precipitating Electron Data Set

**DOI:** 10.1029/2021JA029667

**Published:** 2021-12-19

**Authors:** Joshua M. Pettit, Cora E. Randall, Ethan D. Peck, V. Lynn Harvey

**Affiliations:** ^1^ Laboratory for Atmospheric and Space Physics University of Colorado Boulder CO USA; ^2^ Department of Atmospheric and Oceanic Sciences University of Colorado Boulder CO USA; ^3^ Tamr Cambridge MA USA

**Keywords:** particle precipitation, electrons, middle atmosphere, WACCM, NOx, ozone

## Abstract

The work presented here introduces a new data set for inclusion of energetic electron precipitation (EEP) in climate model simulations. Measurements made by the medium energy proton and electron detector (MEPED) instruments onboard both the Polar Orbiting Environmental Satellites and the European Space Agency Meteorological Operational satellites are used to create global maps of precipitating electron fluxes. Unlike most previous data sets, the electron fluxes are computed using both the 0° and 90° MEPED detectors. Conversion of observed, broadband electron count rates to differential spectral fluxes uses a linear combination of analytical functions instead of a single function. Two dimensional maps of electron spectral flux are created using Delaunay triangulation to account for the relatively sparse nature of the MEPED sampling. This improves on previous studies that use a 1D interpolation over magnetic local time or L‐shell zonal averaging of the MEPED data. A Whole Atmosphere Community Climate Model (WACCM) simulation of the southern hemisphere 2003 winter using the new precipitating electron data set is shown to agree more closely with observations of odd nitrogen than WACCM simulations using other MEPED‐based electron data sets. Simulated EEP‐induced odd nitrogen increases led to ozone losses of more than 15% in the polar stratosphere near 10 hPa in September of 2003.

## Key Points

- A new and improved medium energy electron (MEE) precipitation data set is introduced
- Ionization rates based on the new data result in better agreement between observed and modeled reactive odd nitrogen
- WACCM simulations of the 2003 Antarctic winter show significant MEE‐induced stratospheric odd nitrogen increases and ozone decreases

## 1 Introduction

Space weather has important consequences for Earth's middle and upper atmosphere. Solar flares launched from the Sun release copious numbers of high energy photons that cause large ionization increases in the D and E regions of the ionosphere (Donnelly, 1976; Pettit et al., 2018; Tsurutani et al., 2009; Woods et al., 2004). Many strong solar flares are accompanied by coronal mass ejections (CMEs), which are large magnetic storms that eject massive numbers of solar energetic particles (mostly protons) into the heliosphere. When the CME is directed toward the Earth, the solar protons can penetrate into the upper stratosphere and mesosphere, producing reactive odd nitrogen (NO_x_ = N, NO, NO_2_) and odd hydrogen (HO_x_ = H, OH, HO_2_), which cause catalytic destruction of polar ozone (Jackman et al., 2008; Solomon et al., 1981, 1982; Thorne, 1980). In addition, episodic precipitation of medium (30–1,000 keV) and high energy (>1 MeV) electrons from the radiation belts (e.g., Millan & Thorne, 2007) produces NO_x_ and HO_x_ in the upper stratosphere and mesosphere, as does periodic electron precipitation that occurs in conjunction with high speed solar wind streams from coronal holes (Alves et al., 2006; Kozyra et al., 2006; Miyoshi & Kataoka, 2005; Richardson et al., 2006). Both NO_x_ and HO_x_ are destroyed by UV radiation, so their lifetimes are short, on the order of days, under sunlit conditions in the mesosphere (Solomon et al., 1981, 1982). Even in the dark, HO_x_ is highly reactive and has a short lifetime. NO_x_ on the other hand has a lifetime of months in the mesosphere in the absence of sunlight. Under the right meteorological conditions, such as during the polar winter, mesospheric NO_x_ can be transported into the stratosphere, where it then participates in the catalytic destruction of ozone (e.g., Randall et al., 1998, 2001, 2007).

Although NO_x_ and HO_x_ produced by energetic electron precipitation (EEP) have significant impacts on ozone, precipitating electrons with energies greater than about 30 keV are often excluded in whole atmosphere climate models. This is because available measurements of precipitating electrons have limitations that have historically compromised their application. The work presented here showcases a new data set of precipitating electron fluxes that addresses these limitations. It is based on measurements from the series of medium energy proton and electron detector (MEPED) instruments, which provide the longest available global observational data set of trapped and precipitating electrons. First generation MEPED instruments date back to 1979 as part of the Space Environment Monitor (SEM) platform that flew aboard National Oceanic and Atmospheric Administration (NOAA) satellites. Codrescu et al. (1997) incorporated SEM MEPED electron fluxes in simulations with the Thermosphere Ionosphere Mesosphere Electrodynamics General Circulation Model. They reported significant production of HO_x_ and NO_x_ by the precipitating electrons, and consequent ozone decreases of 27%. At the time, it was not known that the MEPED electron channel data were compromised by proton contamination, particularly during solar proton events (SPEs). Nonetheless, this study pointed out the importance of EEP for middle atmosphere chemistry. The second generation SEM/2 instruments had an improved design, but cross contamination of protons in the electron channels persisted. Various methods have been developed to remove the proton contamination (Lam et al., 2010; Peck et al., 2015; Rodger, Carson, et al., 2010; Rodger, Clilverd, et al., 2010), and several data sets that use proton‐corrected second generation SEM/2 data have now been created (Asikainen & Ruopsa, 2019; Lam et al., 2010; Nesse Tyssøy et al., 2016; Peck et al., 2015; Pettit et al., 2019; van de Kamp et al., 2016, 2018). Modeling studies with these precipitating electron data sets have shown the importance of medium energy electrons (30–1,000 keV) on chemistry of the middle atmosphere (Andersson et al., 2018; Nesse Tyssøy et al., 2016; Pettit et al., 2019; Smith‐Johnsen et al., 2018; Verronen et al., 2015). An outstanding question is whether precipitation of electrons with energies greater than 30 keV results in a larger cumulative effect on the atmosphere than precipitation of solar protons or auroral electrons.

Model‐measurement comparisons shown below indicate that ionization rates based on the new precipitating electron fluxes presented here result in better agreement between observed and simulated NO_x_ than when other MEPED‐based data sets are used. For simplicity, this new EEP data set is hereinafter referred to as the MEPED precipitating electron (MPE) data set. As explained in more detail below, the MPE data set is based on the MP15 data set described in Pettit et al. (2019), but has the following key differences: (a) A new mapping method better accounts for sparse spatial sampling by the MEPED instruments, so the MPE data set better represents regions of intense EEP. (b) Error correction and inclusion of a noise floor have eliminated artifacts that existed in the MP15 data set, and have improved the accuracy of the spectral flux calculations. (c) Inclusion of monthly averaged electron flux maps during SPEs, when MEPED measurements are unreliable, fills in data gaps during SPEs. Section 2 discusses the construction of the MPE data set and improvements over the MP15 data set used in Pettit et al. (2019); it also shows comparisons of the MPE and MP15 electron fluxes. Comparisons of ionization rates from the MPE, MP15, and Coupled Model Intercomparison Project Phase 6 (CMIP6; Matthes et al., 2017; van de Kamp et al., 2016) precipitating electron data sets are shown in Section 3. This section also describes results of WACCM simulations using the MPE, MP15, and CMIP6 data sets, with a focus on NO_x_ and ozone in the mid‐ and high‐latitude stratosphere and mesosphere during 2003. Section 4 presents a summary and conclusion.

## 2 Data and Methods

### 2.1 MPE Data Set

**Figure 1:**
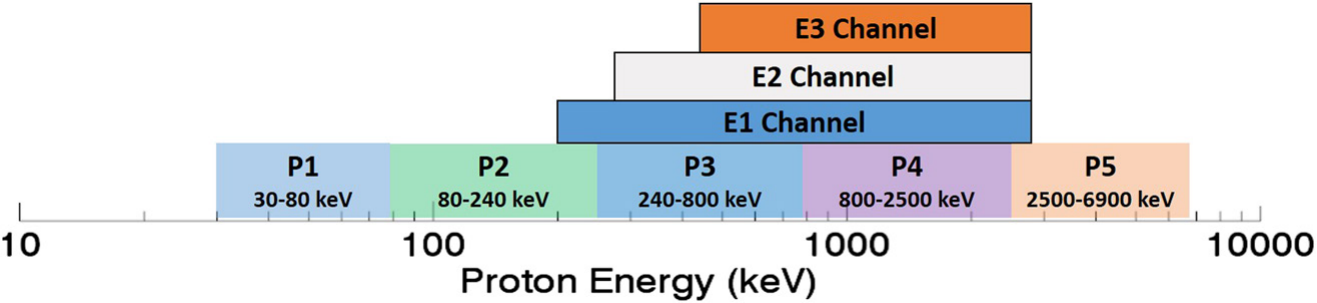
Schematic to show proton contamination in the MEPED electron channels. Colored boxes on the bottom row indicate the MEPED proton channels P1–P6, with their energy ranges. Colored boxes above indicate the three electron channels (labeled E1, E2, E3) that suffer from proton contamination. The horizontal axis refers only to proton energy, not to energies of the electron channels (see the text).

The MPE data set presented here is based on SEM/2 MEPED data and is an improvement over the MP15 data set presented in Pettit et al. (2019). The SEM/2 MEPED instruments, which are described in detail by Evans and Greer (2006) and Green (2013), are onboard the NOAA Polar‐orbiting Observational Environmental Satellites (POES) numbers 15 through 19 as well as all three European Space Agency (ESA) MetOp satellites. This gives the MPE data set a date range from late 1998 through the present day; date ranges for each satellite can be found in Table 1. The MEPED instruments have two telescopes for proton detection and two telescopes for electron detection. Each pair of telescopes includes one that points toward the zenith with a 9° offset (“0‐degree detector”) and one that points in the anti‐ram direction with a 9° offset (“90‐degree detector”); both telescopes have a 30° field of view. As depicted in Figure 1, the proton telescopes have five broadband channels within the energy range from 30 to 6,900 keV, and a P6 channel that detects protons with energies >6,900 keV. The P6 channel is also sensitive to relativistic electrons and can be used as a proxy for electrons with energies greater than ∼700 keV in the absence of protons (Yando et al., 2011). The electron telescopes contain three, overlapping broadband channels coined E1 – E3 that nominally detect electrons with energies of 30–2,500, 100–2,500, and 300–2,500 keV, respectively. The MPE data set includes the P6 channel as a virtual E4 channel that measures electrons with energies >∼700 keV in the absence of protons. The raw data from MEPED are proton and electron count rates that are sampled every other second; these raw data are then averaged every 16 s.

**Table 1:** All Satellites That Have SEM/2 Medium Energy Proton and Electron Detector Instruments, and Their Dates of Operation

Satellite	Data window
NOAA‐15	1998‐07‐01–present
NOAA‐16	2001‐01‐10–2014‐06‐06
NOAA‐17	2002‐07‐12–2013‐04‐10
NOAA‐18	2005‐06‐07–present
NOAA‐19	2009‐02‐23–present
MetOp‐01	2012‐10‐03–present
MetOp‐02	2006‐12‐03–present
MetOp‐03	2019‐01‐01–present

*Note*. Some data gaps exist within the data window.

The end product of the MPE data set is global electron flux maps that include energies from 27 keV up through 8.9 MeV. The MPE data are produced with the following steps: (a) find and remove the proton contamination from the electron channels; (b) calculate the best fit spectrum from the electron channels to convert from count rates to electron fluxes; (c) create electron flux maps at 27 energy intervals using the previously calculated electron fluxes. The resulting flux maps are converted to ionization rates for modeling studies, an example of which will be presented below. Note that the current version of the MPE data set does not include a correction for radiation damage to the older MEPED instruments (Asikainen et al., 2012).

Both the proton and electron MEPED telescopes were designed to prevent cross contamination. The proton telescopes contain cobalt magnets, which bring incident electrons into an aluminum bin to prevent false proton detections. The electron telescopes include nickel‐foil covers to prevent low‐energy protons from entering and being counted as electrons. Unfortunately, this does not prevent protons of certain energies and certain incident angles from producing false detections in the electron channels (Evans & Greer, 2006). The amount of contamination in the electron channels can be small, but varies and requires correction. The method used to correct for proton contamination in the electron channels for the MPE data set follows Peck et al. (2015), and is explained below.

**Figure 2:**
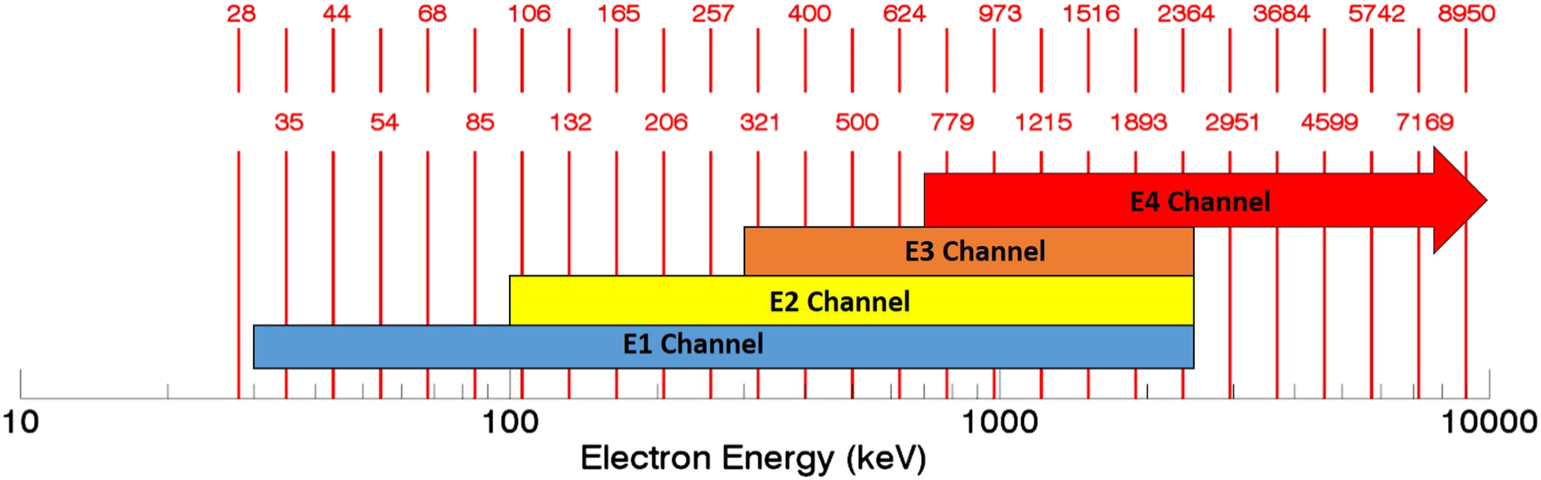
Energy ranges of the four integral electron channels (colored boxes). The red vertical lines indicate the midpoints of the logarithmic energy bins used to calculate differential electron flux spectra for the MPE data set.

There is not a one‐to‐one correspondence between the energies of the protons that are measured by each proton channel and the protons that contaminate each electron channel. Figure 1 demonstrates this by depicting the five broadband proton channels along with the corresponding electron channels that they contaminate. Note that the horizontal axis in Figure 1 pertains only to proton energies; energy ranges of the electron channels are shown in Figure 2. As is evident from Figure 1, the E1 channel is contaminated by protons that range in energy from about 200–3,000 keV; the E2 channel is contaminated by protons that range in energy from ∼300–3,000 keV, and the E3 channel is contaminated by protons that range in energy from ∼500–3,000 keV. Therefore, determining the proton contamination in each of the electron channels requires first calculating the differential proton flux spectrum from the measured proton count rates in the P1‐P5 channels. This is accomplished by first converting the count rates to spectrally integrated fluxes using the geometric factors given in Yando et al. (2011), and then fitting these fluxes to a linear combination of four different analytic functions (power law, energy exponential, single relativistic Maxwellian, double relativistic Maxwellian), as described by Peck et al. (2015). Assuming a linear combination of multiple functions improves the spectral fit to the proton data compared to methods that assume just a single function. Once the differential proton flux is calculated, observed count rates in each of the electron channels are corrected for proton contamination following Peck et al. (2015). This is performed by using the P1‐P5 proton channels to compute a differential proton flux, which is then integrated over the energy ranges that contaminate the various electron channels (see Figure 1). The proton response functions from Yando et al. (2011) are used to convert the fluxes back into proton count rates, which are subtracted from the E1‐E3 electron channel count rates.

The corrected count rates in each electron channel are used to calculate the differential electron flux spectrum. We first apply ideal geometric factors from Evans and Greer (2006) to convert the count rates into broadband electron fluxes in each channel. Following specifications from van de Kamp et al. (2016) to account for the MEPED noise floor, electron fluxes in all three electron channels are set to zero if the E1 channel flux is less than 250 electrons cm^−2^ sr^−1^ s^−1^; note that this is a change from MP15, for which the noise floor correction was applied only after the differential electron flux spectrum was calculated. After the noise floor correction we derive the differential electron flux spectrum from the broadband electron fluxes, by assuming a functional fit, analogous to the calculation of the differential proton flux spectrum described above. Logarithmically spaced energy bins ranging from 28 to 8,950 keV are specified for the differential spectra (see Figure 2). The extension from a high‐energy limit of 1 MeV used in previous studies to 10 MeV used here is made possible by including a virtual “E4” channel, which is derived from the P6 channel. As mentioned previously, in the absence of protons, the P6 channel provides a measure of high‐energy electrons from the radiation belts (Peck et al., 2015; Rodger, Carson, et al., 2010; Rodger, Clilverd, et al., 2010; Yando et al., 2011). The P6 data can thus be used as an additional electron channel. Figure 2 displays the energy ranges of the four electron channels as well as the logarithmically spaced energy bins of the resulting differential electron flux spectra. Vertical red lines denote the midpoint of the energy bins, with the energy corresponding to each line denoted in red (the text for every other bin is offset for clarity). Analogous to the calculation of the proton spectrum, a linear combination of power law, energy exponential, single relativistic Maxwellian, and double relativistic Maxwellian functions is used to compute the differential electron flux spectrum.

**Figure 3:**
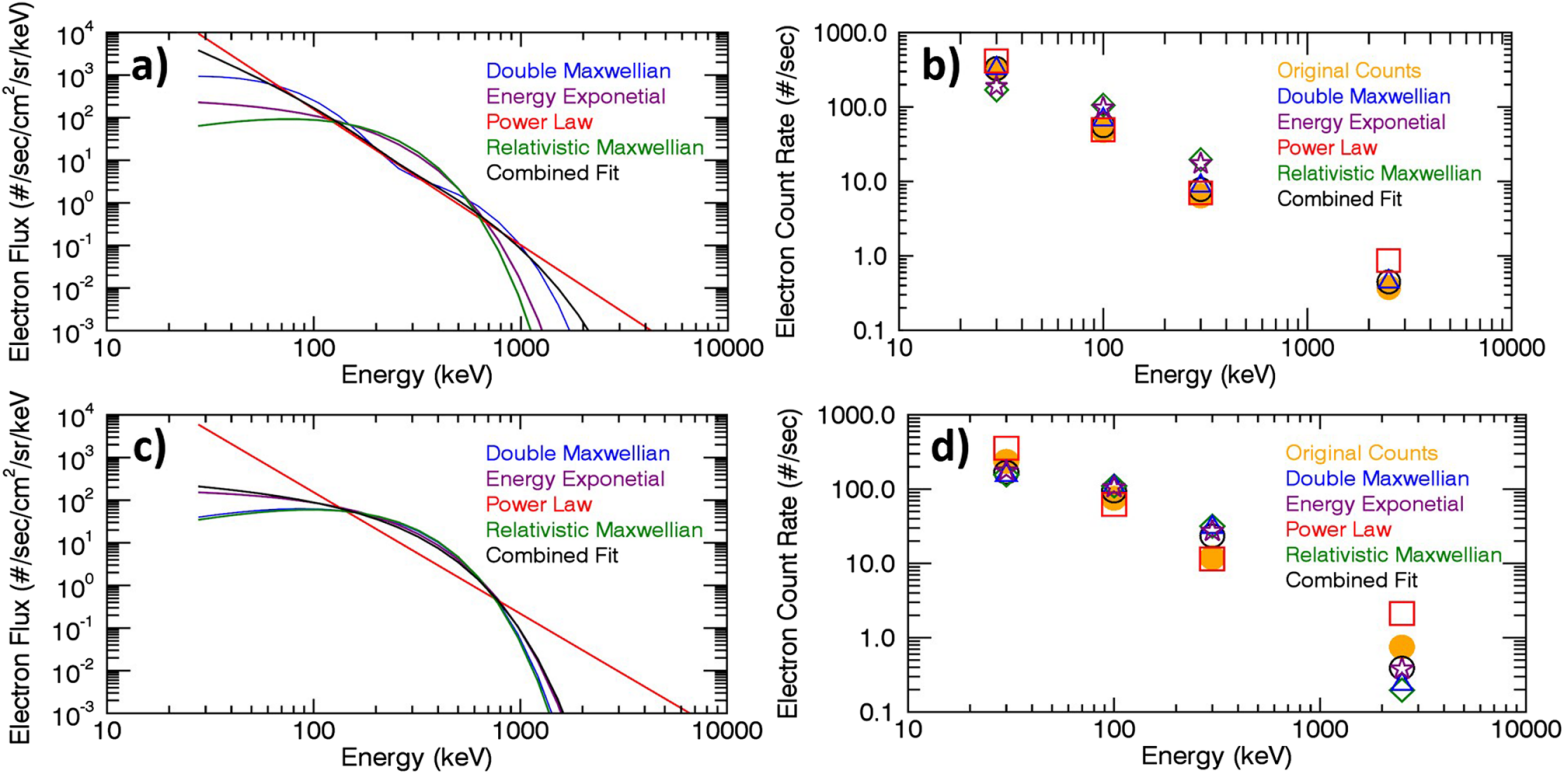
Comparison of differential electron flux spectra (a), (c) calculated from NOAA15 MEPED measurements of electron count rates during one observation on 13 May 2003 at L‐shell 6.19. Panels (b) and (d) show the observed (corrected) count rates (orange dots) as well as the count rates backed out from an inverse analysis of the spectra in panels a and (c) The top panels (a), (b) show results for the 0° telescope and the bottom panels (c), (d) show results for the 90° telescope. The “combined fit” spectrum is used for the MPE data set.

A representative example of the differences between the various single‐function fits and the multifunction combined fit used for the MPE data set is presented in Figure 3. This figure shows results for a single NOAA15 observation on 13 May 2003 at L‐shell 6.19. The left‐hand panels show the various spectra used to make the combined fit as well as the combined fit spectrum itself. The right‐hand panels display the corresponding count rates for each spectral fit, as well as the measured count rates (corrected for proton contamination; orange dots). The count rates corresponding to the various spectra are derived from the differential flux using a model that inverts each spectrum back to count rates in the four channels. The goal of the combined fit spectrum is to minimize the difference between the measured and derived count rates over all four channels. While this figure only displays a single measurement, it demonstrates large differences between the individual spectra and shows that count rates derived from the combined fit spectrum are closest to the measured count rates.

**Figure 4:**
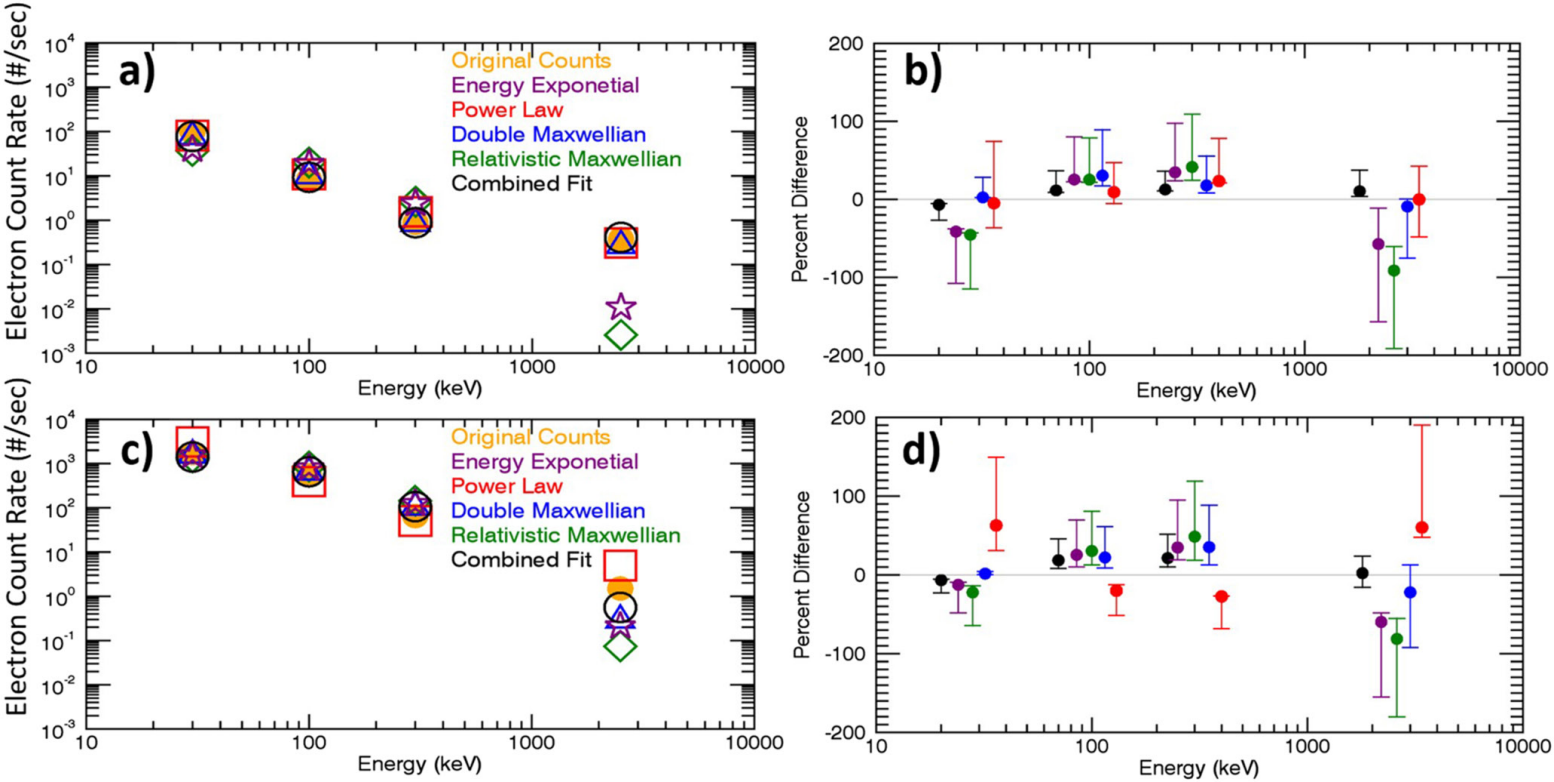
Statistical comparison of observed electron count rates in the E1–E4 channels (“Original Counts”), corrected for proton contamination, to count rates backed out from various spectral fits. Left panels (a), (c) show median count rates in each electron channel for the 0° degree detector (a) and 90° degree detector (c) for all three satellites carrying MEPED instruments throughout 2003. Orange dots labeled “Original Counts” correspond to the corrected observations; open colored symbols correspond to the spectral fits noted in the legend. Right panels (b), (d) show the percent differences between the backed out count rates and measured count rates as colored dots, colored according to the legend in panels a and (c) Symbols in panels b and d are offset in energy for clarity. The interquartile ranges for each spectrum type are represented by the whiskers.

To evaluate the different spectral fits more generally, the same method is performed using all MEPED measurements taken during 2003 between L‐shells 4 and 8. Figure 4 displays these results for both the 0° telescope (top) and the 90° telescope (bottom). Median count rates within each electron channel, which have been derived from the different spectral fits as mentioned above, are presented in the left panels. Percent differences between the measured count rates and the count rates derived from the various spectra are shown in the right panels. Overall, count rates derived from the combined fit spectrum match the observed count rates better than the count rates derived from any of the individual spectral fits. These results demonstrate that the combined fit yields the smallest median differences from the measured count rates as well as the smallest interquartile range. The combined fit particularly outperforms the single function spectral fits in the E4 channel. The double Maxwellian shows the second smallest percent differences overall and outperforms the combined spectrum in the E1 channel for both detectors. However, it is worse in the E2, E3, and E4 channels. The power law spectrum shows fairly small percent differences for the 0° detector, but differences and interquartile ranges are larger in the 90° detector, particularly for the E1 and E4 channels. The energy exponential and relativistic Maxwellian show the largest percent differences in the 0° detector, with especially large differences and interquartile ranges in the E4 channel. While this is also true in the 90° detector, both show much better results overall, outperforming the power law spectrum, due to improved performance in the E1 channel. To summarize, while individual channels may show a specific fit to be better than the combined fit, Figure 4 shows qualitatively that the combined fit most closely matches the observations when all four channels are considered together.

**Figure 5:**
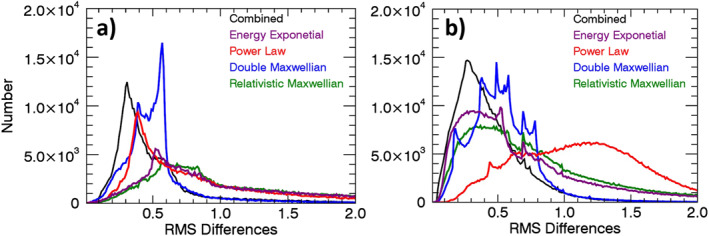
Number distributions of the root mean square (RMS) fractional differences between the count rates backed out from the various spectral fits and the corrected MEPED count rates, for the distribution of measurements in Figure 4. Panel (a) shows results for the 0° degree telescope and panel (b) shows results for the 90° telescope.

Results for all four electron channels are combined quantitatively by calculating the root mean square (RMS) fractional differences between the derived and observed count rates in the E1‐E4 channels. Figure 5 shows the number distributions of the RMS differences for each of the four individual fits as well as for the combined fit, for the measurements included in Figure 4. Fractional differences (calculated‐observed)/observed, were used because the absolute differences in the E1 channel are much larger than the absolute differences in the E4 channel. Histograms of the RMS fractional differences are displayed in Figure 5 for both the 0° (panel a) and 90° (panel b) telescopes using all three available satellites carrying MEPED instruments during 2003. For both telescopes, the combined fit shows the highest number of small RMS fractional differences compared to any of the other individual fits, consistent with the results shown in Figure 4. Likewise, the double Maxwellian shows the second best fit overall. In the 0° telescope, the power law shows the third lowest RMS fractional differences. Both the energy exponential and relativistic Maxwellian show the highest fractional differences in the 0° detector. In the 90° telescope, the energy exponential and relativistic Maxwellian outperform the double Maxwellian fit in terms of higher number of the smallest RMS differences, but both fits have a skewed right‐hand tail that allows for the double Maxwellian to remain the second best overall fit. The power law, which is often used as an assumed spectrum for MEPED data, shows the highest errors in 2003 with the 90° detector. These results give confidence in the combined fit spectrum as the optimum fit to the MEPED data, in agreement with Peck et al. (2015). We do note, however, that this analysis cannot verify the goodness of fit outside the spectral range of the measurements.

As mentioned above, a number of precipitating electron data sets that use MEPED data currently exist. One important differentiating aspect is whether the data sets incorporate one or both of the MEPED electron telescopes. The 0° detector views only part of the bounce loss cone (BLC), whereas the 90° detector views trapped, drift loss cone and BLC electron fluxes (Rodger, Carson, et al., 2010; Rodger, Clilverd, et al., 2010). Electrons in the BLC are considered to be precipitating. Drift loss cone fluxes are considered to be trapped until they reach the South Atlantic Magnetic Anomaly (SAMA), where they will precipitate (Gamble et al., 2008). Trapped electrons may precipitate in the future, but are currently in stable mirroring pathways and are traveling pole‐to‐pole along magnetic field lines. Since it can be difficult to distinguish BLC fluxes from trapped or drift loss cone fluxes in the 90° detector, many available data sets include only the 0° detector, and thus have less information on the flux variation with pitch angle than when both detectors are used. At high latitudes the 0° detector is measuring exclusively BLC flux, at mid‐latitudes a mixture of trapped and BLC flux and at low latitudes exclusively trapped flux. The opposite is true for the 90° detector (Asikainen, 2019). Thus, caution must be used to ensure that only the BLC flux in the detector measurements is being included, otherwise overestimation is likely. When calculating both the drift‐loss cone edge and the BLC edge using data from the International Geomagnetic Reference Field (IGRF) model (Alken et al., 2021), we found that the BLC edge angle is smaller at all magnetic longitudes except within the SAMA. In an attempt to avoid drift‐loss cone flux in our BLC calculations, we integrate only up to the BLC edge, and omit measurements in the SAMA. However, this may not eliminate possible drift‐loss cone flux that may be present in the 90° degree data as shown in Rodger, Carson, et al. (2010) and Rodger, Clilverd, et al. (2010). Previous studies have shown that the 90° detector includes drift‐loss cone flux at a variety of magnetic longitudes and pitch angles. Therefore, despite our attempt to remove the contaminating drift‐loss cone flux in our BLC calculations, the use of the 90° detector may result in overestimation of electron fluxes at mid and lower latitudes where the 90° detector is measuring precipitating electrons.

The MPE data set includes both detectors by assuming a sinusoidal BLC angular distribution with maximum flux at a 90° pitch angle and zero flux at a 0° pitch angle. The sinusoidal function has been used in previous studies (e.g., Gu et al., 2011; Vampola, 1998) and is defined by Equation 1:

(1) $$J_{d}\left(\alpha_{sat}\right)=Asin^{n}\left(\alpha_{sat}\right)$$

Here α_sat_ is the pitch angle at the satellite location, *A* is the amplitude of the sine function and *J*_*d*_ is the measured electron flux. Because of the lack of available pitch angle data, we assume *n* is equal to 1. Upon solving for *A*, the total BLC flux is derived by integrating under the sine curve over the range of BLC pitch angles from 0° to *α*_*BLC*_. Here *α*_*BLC*_ is the maximum BLC pitch angle and is calculated as:

(2) $$sin\left(\alpha_{BLC}\right)=\sqrt{B_{sat}/B_{0}}$$

where *B*_*sat*_ is the magnetic field strength at the satellite altitude and *B*_0_ is the magnetic field strength at 120 km. The magnetic field strength is calculated from the IGRF model (Alken et al., 2021). Both the MPE and MP15 data sets include both the 0° and 90° detectors. On occasion, however, the relative count rates in the two detectors violate the assumption that count rates versus pitch angle should vary as a sine function in the BLC. This occurs when the detector with the lower pitch angle has a higher flux. For these measurements the MPE data set includes only the detector that has the lower pitch angle.

The precipitating electron flux spectra at the MEPED sampling locations are combined into daily, global maps. For each day 27 maps are constructed, 1 for each energy bin (see Figure 2), using a 2° magnetic latitude × 2° magnetic longitude grid. The MPE mapping uses Delaunay triangulation (Delaunay, 1934) to interpolate in space between the measurements on the day in question. In contrast, the mapping method employed for the MP15 data set used a linear interpolation that required between 3 and 7 days of data to populate each 2° × 2° bin. The MP15 maps are thus compromised by electron fluxes that are elevated before or after events due to the multiday averaging. Other MEPED‐based precipitating electron data sets, such as the CMIP6 data set, assume zonal averages on L‐shells, and thus do not capture variations in magnetic longitude. Delaunay triangulation was chosen for the MPE data set because it captures small‐scale features that are obscured by other mapping methods. This is important for geomagnetic substorm activity, which has been suggested to occur frequently (Clilverd et al., 2008; Seppälä et al., 2015).

**Figure 6:**
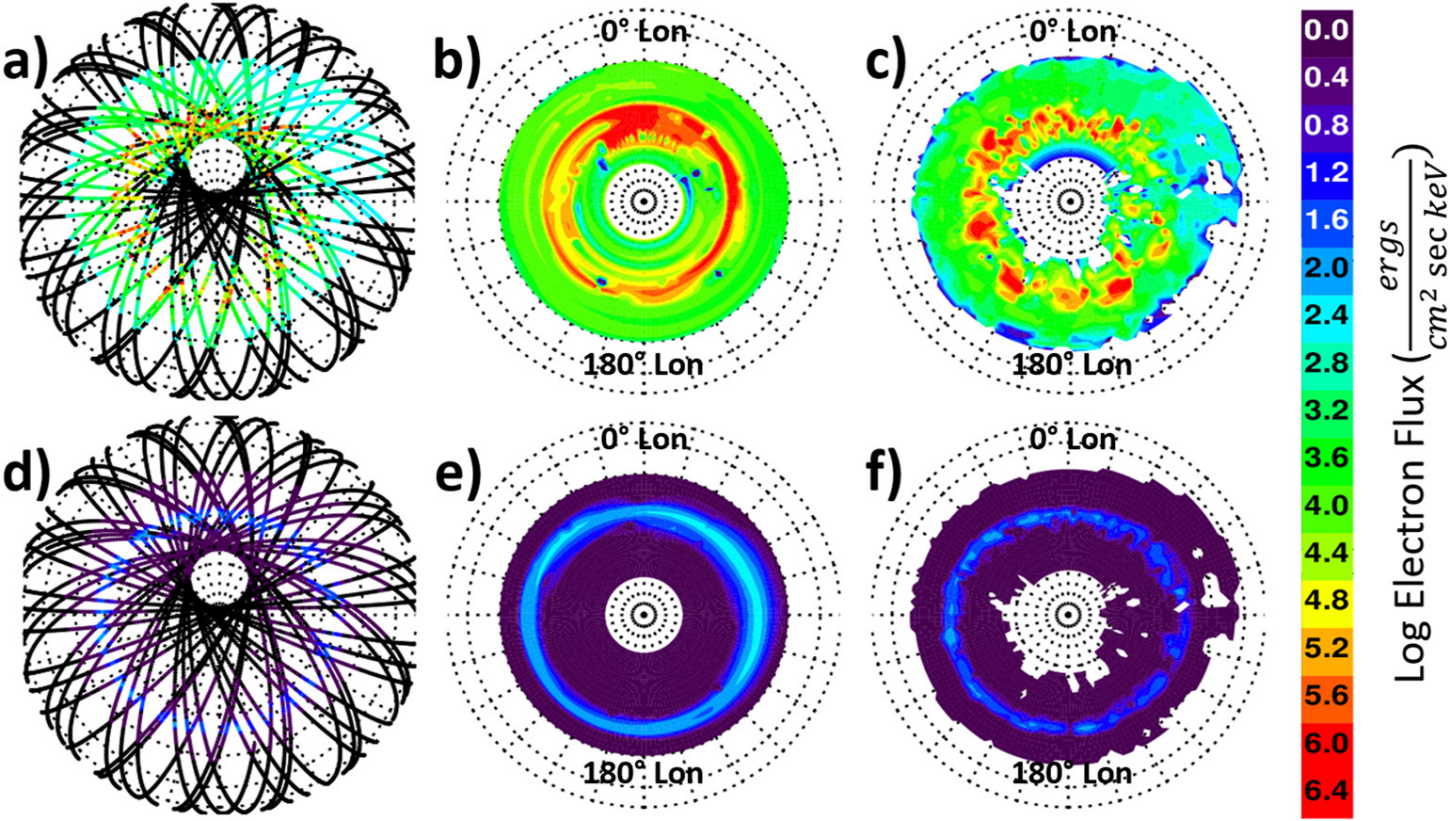
Southern hemisphere polar maps of derived electron flux in the 28‐keV (top) and 779‐keV (bottom) energy bins at the MEPED satellite locations (a), (d) compared to the gridded MP15 (b), (e), and MPE (c), (f) electron flux on 1 May 2003. All maps are shown in geomagnetic coordinates.

Figure 6 compares the derived 28‐keV (top) and 779‐keV (bottom) precipitating electron flux at the satellite locations (a, d) to the gridded flux from the MP15 (b, e) and MPE (c, f) mapping methods for 1 May 2003; all maps are shown in geomagnetic coordinates. To ensure the MP15 and MPE maps include the same data, only 1 day of MEPED data is included in the MP15 map. MEPED observations do not include the geographic pole, which results in the data void regions in panels (a) and (d) that are offset from the geomagnetic pole. Since the interpolation used in the MP15 mapping does not allow for interpolation across the geomagnetic pole, the MP15 maps include a data void region from 83° magnetic latitude to the magnetic pole. The MPE mapping method interpolates across the geomagnetic pole by assuming zonal averages in order to fill in this small region (e.g., in Figures 6c and 6f, near geomagnetic latitude 83°S and 0° magnetic longitude). The comparisons in Figure 6 indicate that because of the Delaunay triangulation, the MPE maps capture more of the observed small‐scale variations than maps based on the MP15 interpolation or L‐shell binning methods. However, some of the small‐scale structures in the MPE maps are caused by artifacts of interpolation over regions with incomplete satellite measurement coverage; there were often gaps in magnetic local time coverage in 2003, when only three MEPED satellites were operating. For most of the MEPED data record at least five satellites are available, so data gaps are smaller. The advantage of using Delaunay triangulation to map the data is that it can capture small‐scale features such as substorm activity that would be averaged out in other data sets. There is a disadvantage in that substorm activity will effectively persist for a day at a time since the maps have 1‐day resolution; in reality, these storms most likely have durations of minutes to hours (Beharrell et al., 2015; Cresswell‐Moorcock et al., 2013; Partamies et al., 2021). On the other hand, it is likely that not all substorms are observed, particularly in a year like 2003 when only three POES satellites were operational. While the triangulation mapping method used in MPE would not be able to capture all substorm activity, its ability to capture some substorm activity is a significant improvement over zonal averaging, which is typically done using MEPED data.

Finally, it is noteworthy that the MPE data set has been adapted for use during SPEs. During SPEs, the MEPED data become unreliable due to significant proton contamination. Therefore, most EEP data sets remove days when SPEs occur. The MPE data set instead uses monthly averaged electron flux maps for days when SPEs occur. This is done by taking the maps from the 15 days before and the 15 days after the day the SPE occurs and averaging them together to produce a map for the day of the SPE. When multiple SPE days occur within the monthly time frame, those days are removed before the average is calculated. This is important since SPEs occur during times of high geomagnetic activity, so removing the SPE days from the data set would cause the electron precipitation to be less accurate during these days. By setting the precipitating electron flux to monthly average values instead of removing them, the MPE data set reduces this inaccuracy.

### 2.2 Model and Simulations

In this work the MPE, MP15, and CMIP6 data sets are evaluated using simulations with the National Center for Atmospheric Research Whole Atmosphere Community Climate Model (WACCM), version 6 (Gettelman et al., 2019). WACCM is the high‐top version of the Community Atmosphere Model (CAM), both of which are part of the Community Earth System Model (CESM) (Gent et al., 2011; Hurrell et al., 2013; Marsh et al., 2013). WACCM6 is run as part of CESM version 2 with a horizontal resolution of 0.95° latitude by 1.25° longitude. The Specified Dynamics version of WACCM6 used here has 88 hybrid sigma pressure levels that extend from the surface to approximately 5.1 × 10^−6^ hPa (∼140 km). WACCM6 includes a comprehensive set of chemical constituents, and chemical processes that are based on previous versions of WACCM (e.g., Kinnison et al., 2007). Particularly relevant to the current work is the addition of D‐region chemistry, which adds negative ion and water cluster ion chemistry to improve the accuracy of chemistry in the mesosphere (Verronen et al., 2016). Inclusion of D‐region chemistry adds 15 positive ions and 21 negative ions to the standard chemistry module.

The solar forcing in WACCM6 uses recommendations from CMIP6, which are described in detail in Matthes et al. (2017). It is important to note that the electron forcing from CMIP6 is designed for long‐term climate simulations and not necessarily for seasonal studies. However, since it is currently the default MEE data set for WACCM6, we chose to use it for comparisons with MPE. SPEs are included using methods from Vitt and Jackman (1995), and the ionization rates from the SPE parameterization have hourly temporal resolution. Auroral electrons are parameterized using Roble and Ridley (1987), as described in Marsh et al. (2007). We use methods from Fang et al. (2008) to compute medium energy electron (MEE) ionization rates, which requires knowledge of the background atmosphere temperature and neutral density. For the CMIP6 data, ionization rates were computed offline using an atmospheric model described in Picone et al. (2002). A complete description of the CMIP6 electron ionization rates can be found in van de Kamp et al. (2016). For the MP15 and MPE data, ionization rates were computed using the background atmosphere from the model. Using the model atmosphere slightly improves the accuracy of the ionization rates within the model simulations (Fang et al., 2008). The ionization rate calculations are dependent on atmospheric temperatures and densities. Thus, the more accurate the temperature and densities are the more accurate the resulting ionization rates will be. Although both the MP15 and MPE data sets include fluxes of electrons with energies greater than 1 MeV, these fluxes were excluded in the ionization rate calculations in accordance with the upper limit of the Fang et al. (2008) electron parameterization.

Four WACCM simulations were conducted for the time period from 1 January 2003 through 30 September 2003. In all simulations the Modern‐Era Retrospective Analysis for Research and Applications (MERRA) reanalysis data (Molod et al., 2015) was used to nudge the model to observed meteorology. A baseline simulation included auroral electron precipitation but no higher energy (>30 keV) electron precipitation. The three other simulations included MEE ionization derived from the CMIP6, MP15, and MPE data sets, respectively.

## 3 Results and Discussion

**Figure 7:**
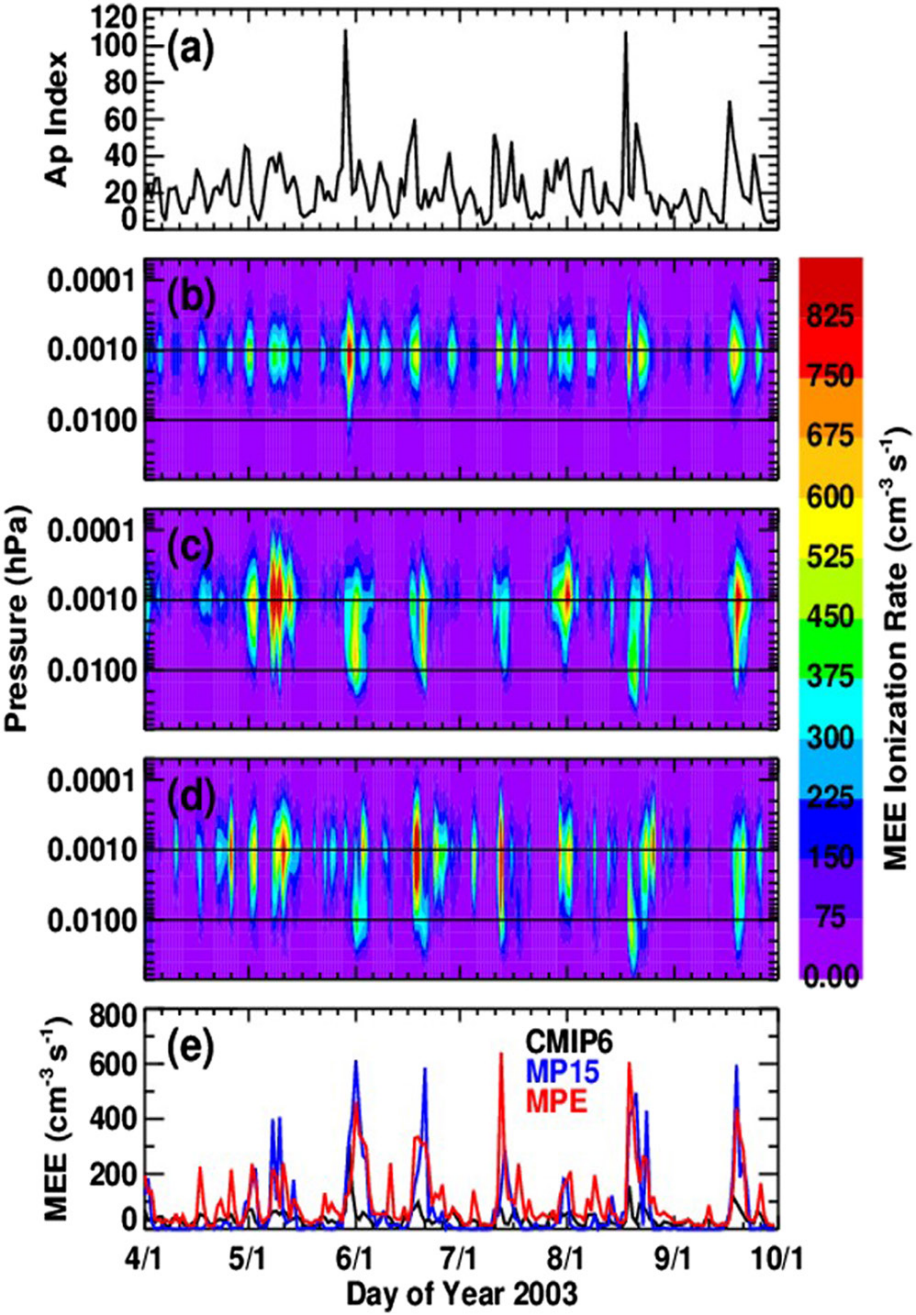
Daily ionization rates in April through September 2003, interpolated to the WACCM geographic grid and averaged from 40° to 90°S, from the CMIP6 (b), MP15 (c), and MPE (d) WACCM simulations. Horizontal lines at 0.001 and 0.01 hPa are for guidance. Panel (e) compares ionization rates at 0.01 hPa from the three simulations. The Ap index, according to which CMIP6 is parameterized, is shown in panel (a).

Figure 7 shows the Ap index from April through September 2003 along with daily ionization rates from CMIP6, MP15, and MPE, which have been interpolated to the WACCM geographic grid and averaged from 40° to 90°S. The timing of the ionization enhancements is similar in all three cases, except that CMIP6 shows slightly earlier increases during high geomagnetic activity. This is because CMIP6 ionization rates are parameterized according to the Ap index, so the two are by definition very well correlated. MEPED count rates tend to be delayed slightly relative to the Ap index, so both the MP15 and MPE ionization rate variations are often delayed relative to CMIP6. The differences in altitude range and in peak ionization altitudes shown in Figure 7 are due to key differences between the data sets. First, both MP15 and MPE use the combined spectral fits, which allow for a larger variation in electron fluxes at higher energies than using a single spectral fit alone. This point is compounded by the inclusion of the 90° degree detector, which tends to have higher count rates at higher energies than the 0° degree detector. CMIP6 utilizes a power law for its fitting of the channels, and a smoothing function is applied to both the latitude and the Ap index, which would limit both the altitude variation and the peak ionization altitude. As a result, both the MP15 and MPE ionization rates have a much wider range of altitudes than the CMIP6 rates and a wider range of peak ionization altitudes. Maximum ionization rates often occur near 0.001 hPa, where the mean rates from 1 April through 30 September are 210, 230, and 224 ion pairs cm^−3^ s^−1^ for the CMIP6, MP15, and MPE simulations, respectively. Ionization occurs all the way down to 0.01 hPa more often in the MPE data set than in either the MP15 or CMIP6 data sets, leading to more ionization at this altitude in the MPE data set. This is shown in Figure 7e, which compares daily ionization rates at 0.01 hPa from all three data sets. Mean ionization rates at 0.01 hPa from April through September are 36, 66, and 99 ion pairs cm^−3^ s^−1^ for the CMIP6, MP15, and MPE simulations, respectively. The larger mean MPE ionization rate at 0.01 hPa is attributed to the MPE calculation capturing more high‐energy electron precipitation than the CMIP6 or MP15 methods. Column‐integrated ionization rates (not shown) indicate that the MPE calculation leads to more ionization overall than the CMIP6 or MP15 calculations. Mean column‐integrated ionization rates over the time period shown are 8.5 × 10^9^, 1.9 × 10^10^, and 2.2 × 10^10^ ion pairs cm^−2^ s^−1^ for the CMIP6, MP15, and MPE simulations, respectively. That overall MPE ionization rates are higher than MP15 rates is attributed to the combined effects of the changes incorporated into the MPE electron flux mapping method, consideration of the noise floor, and accounting for MEE during SPEs. The deeper penetration altitude and larger column‐integrated magnitude of MPE ionization suggest that middle atmosphere effects of particle precipitation will be more significant when calculated with the MPE data set, consistent with the WACCM model results discussed next.

**Figure 8:**
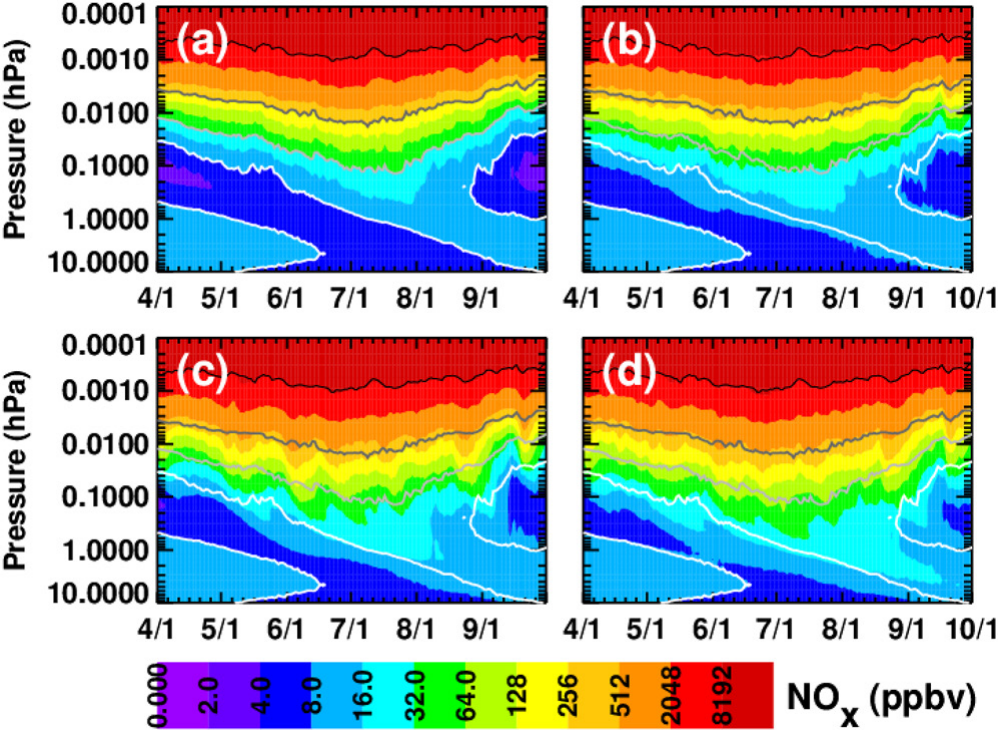
WACCM NO_x_ mixing ratios, averaged daily over latitudes from 40° to 90°S, from the (a) baseline, (b) CMIP6, (c) MP15, and (d) MPE simulations. For guidance, black, dark‐gray, light‐gray, and white contour lines denote the baseline NO_x_ isolines at 8,192, 256, 32, and 8 ppbv, respectively.

To show how the different MEE ionization rates affect NO_x_, Figure 8 displays the NO_x_ mixing ratios from the WACCM baseline simulation and the three MEE simulations for the same time period as in Figure 7, averaged over latitudes from 40° to 90°S. All four simulations clearly show NO_x_ descent throughout the winter, indicative of the well‐known indirect effect of energetic particle precipitation (EPP) (Randall et al., 2006, 2007). To aid in visual comparisons, contour lines in black, two shades of gray, and white have been superimposed on all panels in Figure 8 to denote the baseline simulation NO_x_ isolines at 8,192, 256, 32, and 8 ppbv. More NO_x_ descends to lower altitudes in the MEE simulations than in the baseline simulation, with the MPE simulation showing the most NO_x_ reaching 10 hPa. For example, median NO_x_ differences between the MEE simulations and the baseline (MEE minus baseline) at 0.01 hPa (∼70 km) from May through August, the polar winter time period of greatest interest for the EPP indirect effect at this altitude, are 24% for CMIP6, 64% for MP15%, and 99% for MPE. The corresponding differences at 1 hPa (∼50 km) from June through August, the period of greatest descending NO_x_ enhancements at this altitude, are 27%, 81%, and 131%.

**Figure 9:**
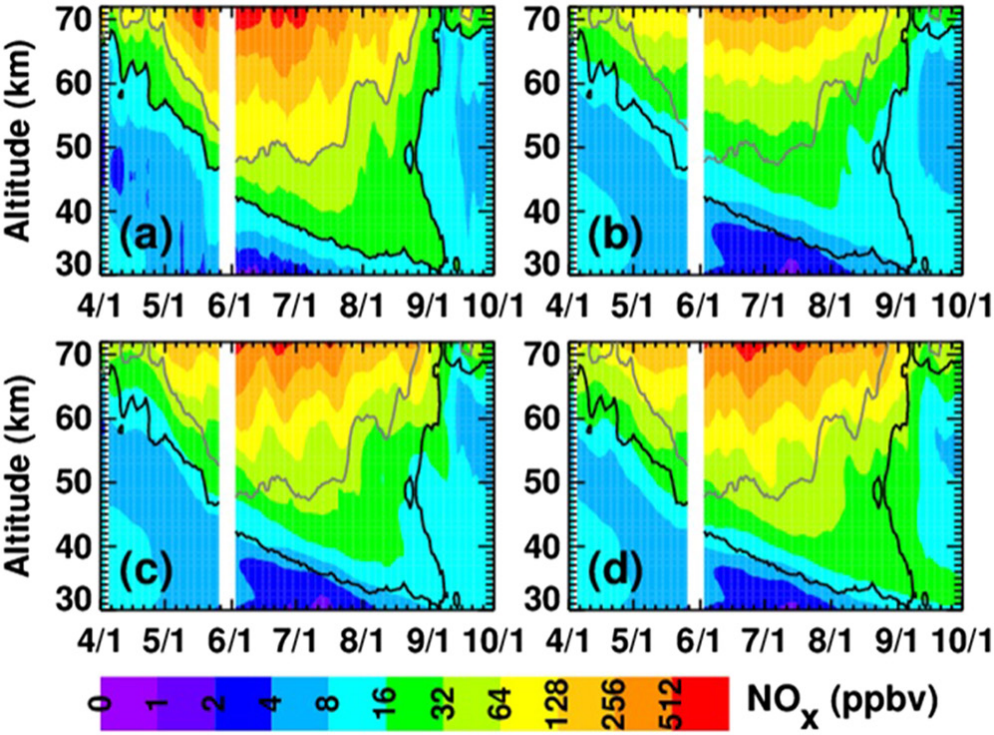
Five‐day running mean NO_x_ mixing ratios averaged over latitudes from 70° to 90°S, from MIPAS (a), CMIP6 (b), MP15 (c), and MPE (d). WACCM simulations are sampled at the times and locations of the MIPAS measurements. The gray and black contour lines indicate the 64 and 16 ppbv MIPAS NO_x_ isolines, respectively. White regions indicate missing MIPAS data or regions where MIPAS errors exceed 200%.

To evaluate the veracity of the MEE input data sets, Figure 9 compares NO_x_ mixing ratios in the three model simulations that include MEE to NO_x_ observations made by the Michelson Interferometer for Passive Atmospheric Sounding (MIPAS) (Fischer et al., 2008; Funke et al., 2011). MIPAS measured daily global NO and NO_2_ from 2002 to 2012 from the surface through approximately 75 km (Funke, López‐Puertas, Stiller, & von Clarmann, 2014; Funke, López‐Puertas, Holt, et al., 2014). The panels in Figure 9 show polar cap (70° to 90°S) average NO_x_ mixing ratios as a function of altitude and time; the model simulations are sampled at the locations and times of the satellite measurements. The black and gray contour lines indicate the MIPAS 16 and 64 ppbv NO_x_ mixing ratio isolines to help guide the reader to the important regions of NO_x_ descent. White areas indicate missing or erroneous MIPAS data, which we define as data for which the published errors exceed 200%. Figure 9a shows MIPAS measurements of NO_x_, while panels (b)‐(d) show NO_x_ in the CMIP6, MP15, and MPE simulations, respectively. Qualitatively, it is clear from Figure 9 that the amount of NO_x_ descending during the SH 2003 winter is larger in the MPE simulation than in either the MP15 or CMIP6 simulations, and that inside the main region of descending NO_x_, the MPE results agree best with MIPAS.

More quantitatively, median differences (WACCM minus MIPAS) from May through August at 70 km (∼0.01 hPa) are −43% (−113 ppbv) for CMIP6, −18% (−51 ppbv) for MP15%, and −3% (−6 ppbv) for MPE. As described above and shown in Figure 7, 0.01 hPa is near the bottom limit of the altitude range where MEE ionization is occurring. Thus, although MIPAS measurements were not made at higher altitudes closer to the MEE ionization maximum, Figure 9 comparisons suggest that MPE ionization rates in the mesosphere are more realistic than in the other MEPED‐based data sets. We cannot, however, definitively rule out a possible overestimate in MPE ionization above 70 km that is partially counteracted by an underestimate in descent. Similar results pertain to the main region (the so‐called tongue) of descending NO_x_. That is, as expected from the fact that EEP‐produced NO_x_ (EEP‐NO_x_) is largest in the MPE simulation, NO_x_ mixing ratios in the tongue of descending air are also highest in the MPE simulation. Median differences relative to MIPAS at 50 km from June through August, the time period when the tongue is most prominent at 50 km, are −56% (−30 ppbv) for CMIP6, −36% (−17 ppbv) for MP15%, and −12% (−7 ppbv) for MPE. Mean differences at 40 km from June through August are −53% (−12 ppbv) for CMIP6, −37% (−9 ppbv) for MP15%, and −12% (−3 ppbv) for MPE. All of the simulations underestimate the amount of descending NO_x_ relative to MIPAS, but the model‐measurement agreement is best for the MPE simulation.

Outside the main region of descending NO_x_, all simulations, including the baseline (not shown), overestimate NO_x_ mixing ratios in the stratosphere in April. For example, median differences in April at 40 km are +84% (CMIP6), +82% (MP15), and +84% (MPE), or ∼4 ppbv. The similarity in these results suggests that the WACCM high NO_x_ bias in April is not due primarily to MEE, but rather is perhaps related to the model initialization. More variation between the simulations is seen outside the main region of descending NO_x_ in the stratosphere in September. At 40 km the median WACCM‐MIPAS differences in September are −4% (−0.4 ppbv), +9% (+1 ppbv) and +44% (+4 ppbv) for CMIP6, MP15, and MPE, respectively. Possible contributing factors for the high bias in the MPE simulation are too much descent of NO_x_‐rich air in September, and/or too much confinement of the NO_x_‐rich air in the vortex; both factors would result in the MPE simulation having a higher bias than the other simulations. In addition, the underestimate of descending NO_x_ in the CMIP6 and MP15 simulations might have offset the initial high bias in April, leading to a smaller high bias in September.

**Figure 10:**
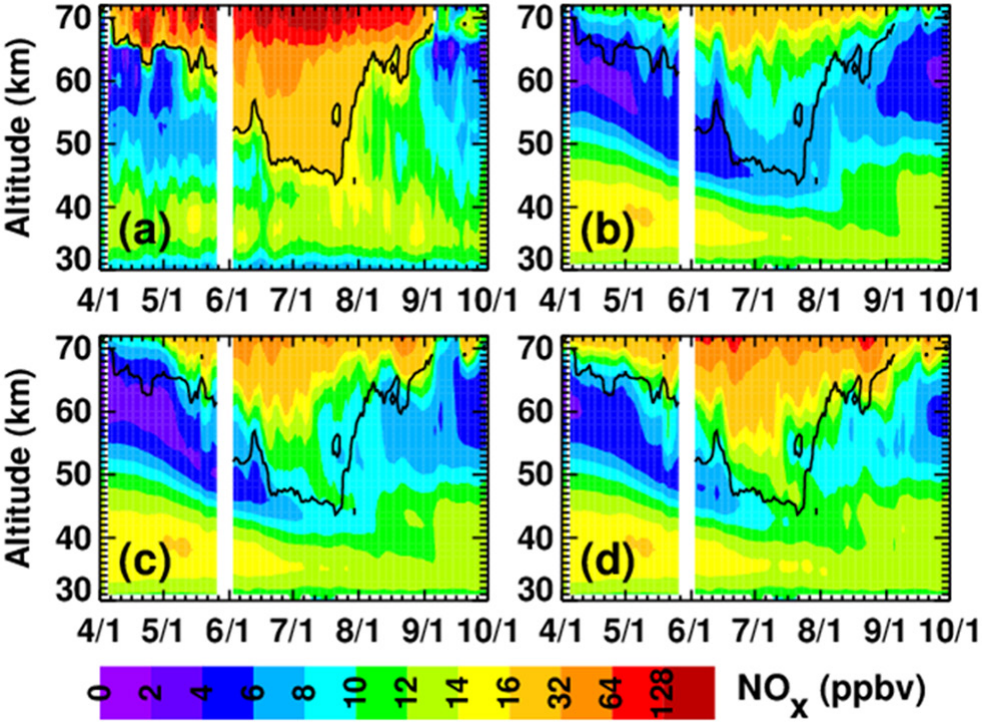
As in Figure 9, but for latitudes from 40° to 50°S; the black contour line denotes the 16 ppbv MIPAS NO_x_ isoline.

As noted in Pettit et al. (2019), MEE affects NO_x_ at mid‐latitudes as well as polar latitudes. This is confirmed in Figure 10, which is analogous to Figure 9, but pertains to latitudes between 40° and 50°S. Relative to MIPAS, all of the simulations underestimate the descending NO_x_ at these latitudes. The underestimate is most severe in the CMIP6 simulation; this is attributed largely to the fact that the CMIP6 ionization rate calculation excludes the MEPED 90° detector, and at these latitudes the 90° detector begins measuring precipitating fluxes and the 0° detector begins measuring trapped fluxes, as explained in Section 2.1 above. This is due to the orientation of the telescopes, which causes the 0° degree detector's pitch angle to decrease with increasing latitude and the 90° detector's pitch angle to increase with increasing latitude. The MP15 and MPE simulations show better agreement than the CMIP6 simulation, but still underestimate NO_x_ mixing ratios inside the tongue. This is true despite the possibility of overestimation of fluxes at these latitudes due to drift‐loss cone contamination. From May through August, median differences (WACCM‐MIPAS) at 70 km are −83% (−79 ppbv) for CMIP6, −77% (−70 ppbv) for MP15%, and −65% (−58 ppbv) for MPE. Median differences from June through August at 50 km are −52% (−7 ppbv), −34% (−5 ppbv), and −27% (−4 ppbv), respectively, for CMIP6, MP15, and MPE. The respective differences at 40 km are −21% (−3 ppbv), −18% (−2 ppbv), and −13% (−2 ppbv). Similar to the polar results shown in Figure 9, NO_x_ mixing ratios in the stratosphere in September are higher than observed by MIPAS in all simulations, with the MPE simulation showing the highest bias. Median differences in September are +12%, +16%, and +21% (1–2 ppbv) for the CMIP6, MP15, and MPE simulations, respectively. Although not as noticeable at latitudes from 70° to 90°S, the simulated NO_x_ increases at 40°–50°S from ∼30 to 45 km in September suggest that the springtime conversion of reservoir odd nitrogen to NO_x_ (e.g., Randall et al., 1998) is contributing to the high bias in all three WACCM simulations.

**Figure 11:**
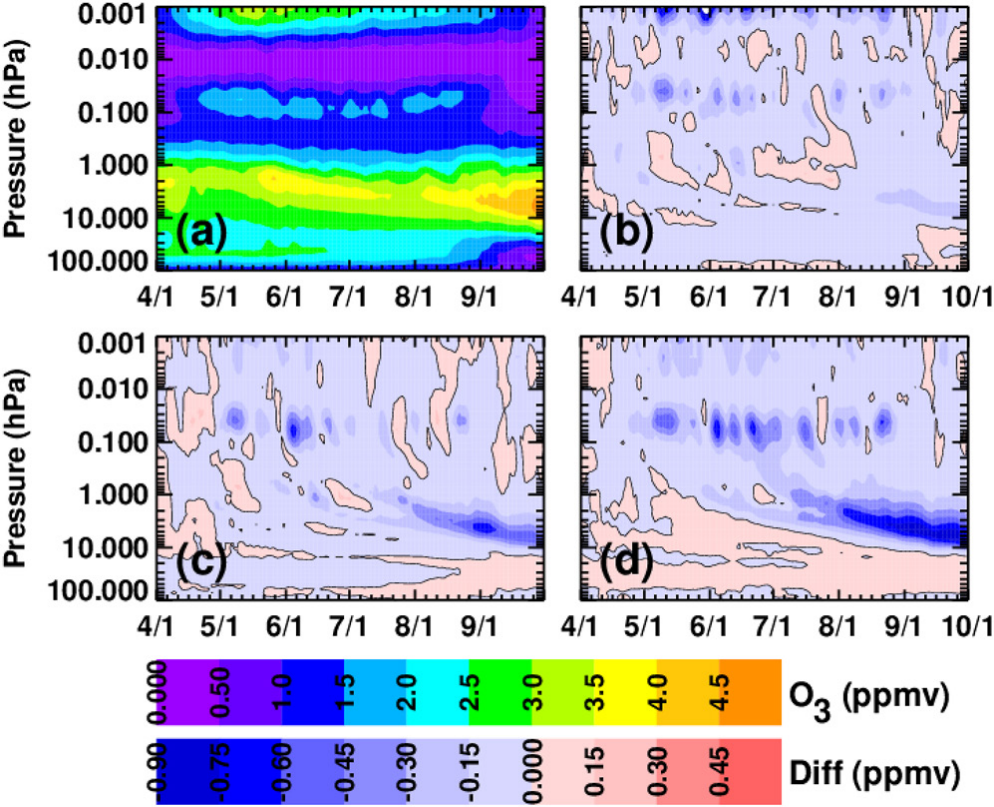
Five‐day running mean O_3_ mixing ratios, averaged over latitudes from 70° to 90°S, from the WACCM baseline simulation (a), and differences between the forced and baseline simulation (forced minus baseline) for CMIP6 (b), MP15 (c), and MPE (d).

Since this work is motivated by the role of NO_x_ in the catalytic destruction of stratospheric ozone, Figure 11 shows the contoured ozone mixing ratios for the baseline simulation (a) along with the ozone mixing ratio differences relative to the baseline for CMIP6 (b), MP15 (c), and MPE (d). In all three MEE simulations there is a region of ozone loss at the tertiary ozone maximum (Marsh et al., 2001) between about 0.1 and 0.05 hPa in May through August, with largest loss in the MPE simulation. In addition, the MP15 and MPE simulations show a region of ozone loss that descends from ∼0.1 hPa in June to 10 hPa by mid‐September. The ozone loss from 0.1 to 0.05 hPa is due to the production of OH from the MEE ionization. Since the MPE data set shows the most ionization out of the three data sets at this level, it also shows the most ozone destruction at this level. At altitudes below 0.1 hPa, the ozone loss is caused by NO_x_ that was produced in the mesosphere by MEE precipitation and then transported into the stratosphere. This interpretation is consistent with the results in Figure 8, which show the descent of NO_x_ from 0.1 to 10 hPa throughout the southern winter. As expected, there is more ozone loss below 0.1 hPa in the MPE simulation than in the other simulations, with a median decrease of >0.75 ppmv (15%–20%) near 10 hPa in September.

## 4 Summary and Conclusions

This study describes a new EEP data set derived from the series of MEPED instruments aboard the NOAA and MetOp satellites. The new data set, which is referred to as the MPE data set, is an improved version of the MP15 data set (Pettit et al., 2019). Improvements include additional MEPED noise floor constraints, modification of the spectral fitting, ignoring one of the telescopes when MEPED count rates violate the assumed sinusoidal variation with pitch angle, substitutions for days with SPEs, and a new mapping procedure. The geomagnetically active time period from April through September of 2003 was investigated to evaluate the MPE ionization rates. Column‐integrated MPE ionization rates are on average about 15% greater than MP15 ionization rates, and more than 2.5 times greater than the MEPED‐based CMIP6 ionization rates (Matthes et al., 2017; van de Kamp et al., 2016). The lower CMIP6 ionization rates relative to the MP15 and MPE rates are attributed partially to the fact that the CMIP6 calculation does not incorporate measurements from the 90° MEPED detector. Another contributing factor is the CMIP6 parameterization versus geomagnetic index, which tends to average out the daily fluctuations that can be seen when using the POES data directly.

To quantify the MEE precipitation effects on the distributions of NO_x_ and ozone, WACCM simulations of the 2003 Antarctic winter were forced with the MPE, MP15, and CMIP6 MEPED‐based EEP ionization rates and compared to a baseline simulation that only included auroral ionization. Consistent with the ionization rate comparisons, more NO_x_ was produced by EEP in the MPE simulation than in the MP15 or CMIP6 simulations. The average NO_x_ mixing ratio increase at latitudes from 40° to 90°S in the mesosphere near 70 km in the MPE simulation was 1.5 times greater than in the MP15 simulation, and four times greater than in the CMIP6 calculation. In all three simulations, EEP‐produced NO_x_ descended throughout the winter. Relative to the baseline simulation without MEE, the MPE simulation showed average NO_x_ increases caused by the descending EEP‐NO_x_ of more than 60% at 10 hPa in September from 40° to 90°S. These NO_x_ increases resulted in median ozone losses in September of ∼0.75 ppmv (15%–20%) in the middle and upper stratosphere. Comparisons of the WACCM results with MIPAS measurements indicate that simulated NO_x_ descending from the mesosphere into the stratosphere at both polar and mid‐latitudes is overall in better agreement with observations when EEP is specified using the MPE data set than the MP15 or CMIP6 data sets. However, in September outside the main tongue of descending EEP‐NO_x_, the MPE simulation overestimates stratospheric NO_x_ mixing ratios more than the other simulations. Determining the cause of this bias is outside the scope of this paper, but it is most likely a combination of model initialization as well as transport and chemical processes. At mid‐latitudes, all three simulations including MP15 and MPE underestimated EEP‐NO_x_ despite the possibility of overestimation from drift‐loss cone flux in the 90° detector. Further quantification of the drift‐loss cone flux will be undertaken in the next version of the MPE data set.

In conclusion, the results described above suggest that coupled chemistry climate model calculations of MEE effects on the middle atmosphere will benefit from incorporation of ionization rates based on the MPE electron flux maps. It is important to note that the MPE‐based ionization rates described in this work, like all other MEPED‐based ionization rates to date, are applicable only for precipitating electrons with energies less than ∼1 MeV. This is because most current ionization rate calculations use the well‐known parameterization created by Fang et al. (2008) to convert from electron flux to ionization rates, and this parameterization is currently limited to electron energies ≤1 MeV. In the future, the use of a new parameterization (Xu et al., 2018) to calculate ionization rates will be explored. Unlike other MEPED‐based data sets, the MP15 and MPE spectral calculations extend the estimates of precipitating electron fluxes to include not only those electrons with energies less than 1 MeV, but also those with energies up to 8.9 MeV. The Xu et al. (2018) parameterization will enable the calculation of ionization rates for these highly relativistic electrons, and thus investigations of how these electrons impact the atmosphere.

## Data Availability

The CMIP6 data set, as well as a metadata description and tools to convert and implement the solar forcing data, can be found at https://solarisheppa.geomar.de/cmip6. The POES MEPED data is available at the National Centers for Environmental Information (NCEI) at https://www.ngdc.noaa.gov/stp/satellite/poes/dataaccess.html. The MP15 electron flux data is available at https://figshare.com/s/fa9196a61db4833bae0c. The MPE electron flux data is available at https://figshare.com/s/c13dd42673bd7f780589 and the data set in its entirety will soon be added to NOAA's National Center for Environmental Information data repository along with SWx TREC (https://lasp.colorado.edu/space-weather-portal/home). Finally, we would like to thank the European Space Agency for use of the MIPAS data and in particular Bernd Funke from the Karlsruhe Institute of Technology for the latest version of the MIPAS data.
